# Genome sequencing reveals existence of SARS-CoV-2 B.1.1.529 variant in Egypt

**DOI:** 10.1186/s43141-022-00352-1

**Published:** 2022-05-11

**Authors:** Ghada Ismail, Hossam Abdelghaffar, Mohamed G. Seadawy, Mostafa F. El-Hosseny, Ahmed F. Gad, Amr Ageez, Ahmed ElShafei, Shereen Saeed Mohammed, Marym Saied Ali, Marwa abd El-Rasoul El-Ashry

**Affiliations:** 1 Clinical Pathology Department, Faculty of Medicine, Ain Shams University, Al-Abbasia PO11588, Cairo, Egypt; 2 Reference Laboratory of the Egyptian University Hospitals (RLEUH), Cairo, Egypt; 3 Department of ENT, Faculty of Medicine, Helwan University, Helwan, Egypt; 4 Supreme Council for University Hospitals, Cairo, Egypt; 5 Biological Prevention Department, Chemical Warfare, 4.5 km Suez-Cairo Rd, Almaza, Cairo, Egypt; 6 Faculty of Biotechnology, MSA University, 6 October City, Egypt; 7 Theodor Belharz Research Institute, Warraq Al Hadar, Egypt

**Keywords:** Omicron variant, Egypt, 21K, genome analysis

## Abstract

**Background:**

Several SARS-CoV-2 variants with increased transmissibility and/or potential immune escape have emerged and rapidly spread worldwide. Ongoing surveillance analyses are performed worldwide to designate new variants of concern (VOC) of coronavirus.

**Main text:**

This report identifies the first Egyptian patient with a confirmed SARS-CoV-2 omicron variant. The patient showed positivity on reverse transcriptase-polymerase chain reaction and full genome sequencing was performed to confirm the variant. The mutations found in the variant were compared with the GISAID reference strain hCoV-19/Wuhan/WIV04/2019. Genome BLAST showed the highest similarity to omicron variants isolated in South Africa. Phylogenetic analysis revealed that the variant belongs to the 21K clade.

**Conclusions:**

The study indicates the importance of information-sharing among global public health partners. Moreover the importance of implementation of full genome sequencing to rapidly identify and track the new SARS-CoV-2 variants.

## Background

The severe acute respiratory syndrome coronavirus 2 (SARS-CoV-2) was first being identified in December 2019. The virus was responsible for massive human morbidity and mortality worldwide [1–3]. In late 2020, several SARS-CoV-2 “variants of concern” emerged that dictate special scrutiny. The new variants include the UK (UK) alpha variant (B.1.1.7), South Africa beta variant (B.1.351), Gamma (B.1.1.28.1), and Delta (B.1.617.2). The new variants have increased transmissibility and/or potential immune escape [4–7].

B.1.1.529 named Omicron variant, was first discovered in Botswana on November 11, 2021. South Africa reported it to the World Health Organization on November 24, 2021, and it was designated as a variant of concern (VOC) on November 26, 2021 [8]. As of 15 December 2021, the Omicron variant has already popped up in around 77 countries with the majority of the cases from the UK, South Africa, and the USA [9]. B.1.1.529 has 32 mutations on the spike protein in comparison with Delta variant (B.1.617.2) that had 9 mutations on the spike protein [10].

Ongoing surveillance analyses are performed worldwide to designate new variants of concern (VOC) of coronavirus. The surveillance is based upon utilizing RT-qPCR techniques for SARS-CoV-2 detection [11, 12], beside the use of next-generation sequencing (NGS) [13, 14]. The continuing surveillance gives the opportunity to track the SARS-CoV-2 genome evolution and variability. Moreover, the use of databases such as GISAID; (https://www.gisaid.org ) that elucidate its genomic characteristics, and Nextstrain; (https://nextstrain.org ) which monitors its spread within the global population.

This report reports the first omicron variant isolated in Egypt. The variant was verified by reverse transcriptase- polymerase chain reaction (RT-PCR), in addition to genomic sequencing and viral phylogenetic analysis.

## Main text

A 67-year-old male patient from Ataka, Suez governorate, Egypt, developed mild respiratory symptoms (fever 38 °C, cough, anosmia, and shortness of breath) on 20, November 2021 and was managed for home isolation with symptomatic treatment namely paracetamol and vitamin C supplements. The patient had no history of travel abroad in the last 6 months. He was vaccinated by Sinopharm (1st dose: 3/4/2021 and 2nd dose 24/4/2021) at the Ministry of Health and Population family Unit, Faisal, Suez governorate. Forty-eight hours before the appearance of patient symptoms he had been exposed to a suspected carrier (his son, who had given home isolation care to his sick aunt that is COVID-19 laboratory-confirmed case.

On the 6th of December, 2021 the patient suffered dyspnea and disturbed level of consciousness and was subsequently admitted to Cleopatra Queens hospital, Cairo. A nasopharyngeal swab was collected on the next day and sent to the Reference laboratory for University Hospitals (RLEUH) for COVID 19 laboratory confirmation. Patient received COVID-19 agreed treatment according to the Egyptian ministry of health protocol in correlation with the patient medical condition. On 16th December, 2021 patient was discharged from the hospital after subsiding all symptoms.

RNA extraction was performed by Microlab Nimbus (Hamilton, USA) and a positive result with Ct value 26 was confirmed using Allplex™ 2019-nCoV Assay (Seegene Technologies, Korea). Variant detection was performed using Allplex™ SARS-CoV-2 Master Assay (Seegene Technologies, Korea), and Bio-Rad CFX 96 platform (Bio-Rad, USA). Full genome sequencing was performed using AviSeq COV19 NGS Library Prep kit (Avicenna, UAE). Next-generation sequencing was performed on Illumina iSeq 100 instrument (Illumina, San Diego, US).

## Results and discussion

The paired-end raw reads with 100X coverage resulting from the sequencing process were quality-filtered and trimmed using Trimmomatic v0.38 [15]. FASTA files were generated using the virus pathogen database and analysis resource (ViPR) [16]. The resulted FASTA genome was analyzed with Pangolin version 3.1.17 with pango LEARN version 2021-12-06 and pango version v1.2.105 (https://cov-lineages.org/resources/pangolin.html, last accessed January 08, 2022). Pangolin determined the lineage for the assembly to be BA.1 with a conflict of 0.0. The variant was identified Omicron GRA (B.1.1.529+BA.*). The sequence has been deposited in the Global Initiative on Sharing All Influenza Data (GISAID) database with accession ID: EPI_ISL_7952324. Insertion of 6 nucleotides and Gap of 36 nucleotides were identified in the assembly when compared to the reference WIV04 sequence. . A total of sixty-seven mutations, compared to the reference WIV04 sequence, were identified in the assembly, thirty-one of them are in the S protein (Table 1).

**Table 1:** List of protein SNPs identified in the isolated variant

Gene	SNPs
S	A67V H69L V70I T95I N211I G339D V367F S371P S371F S373P S375F S477N T478K E484A Q493R G496S Q498R N501Y Y505H T547K D614G H655Y N679K P681H N764K D796Y N856K Q954H N969K L981F D1146D
ORF1ab	K856R K856R F924F F924F A1707A A1707A A2710T A2710T T3255I T3255I P3395H P3395H L3674F L3674F G3676S G3676S I3758V I3758V V4310V V4310V P314L N591N I1566V
ORF3a	T64T
E	T9I
M	D3G Q19E A63T
ORF6	R20R
N	P13L G30G S33G R203K R203R G204R
ORF7b	L18L

**Fig. 1:**
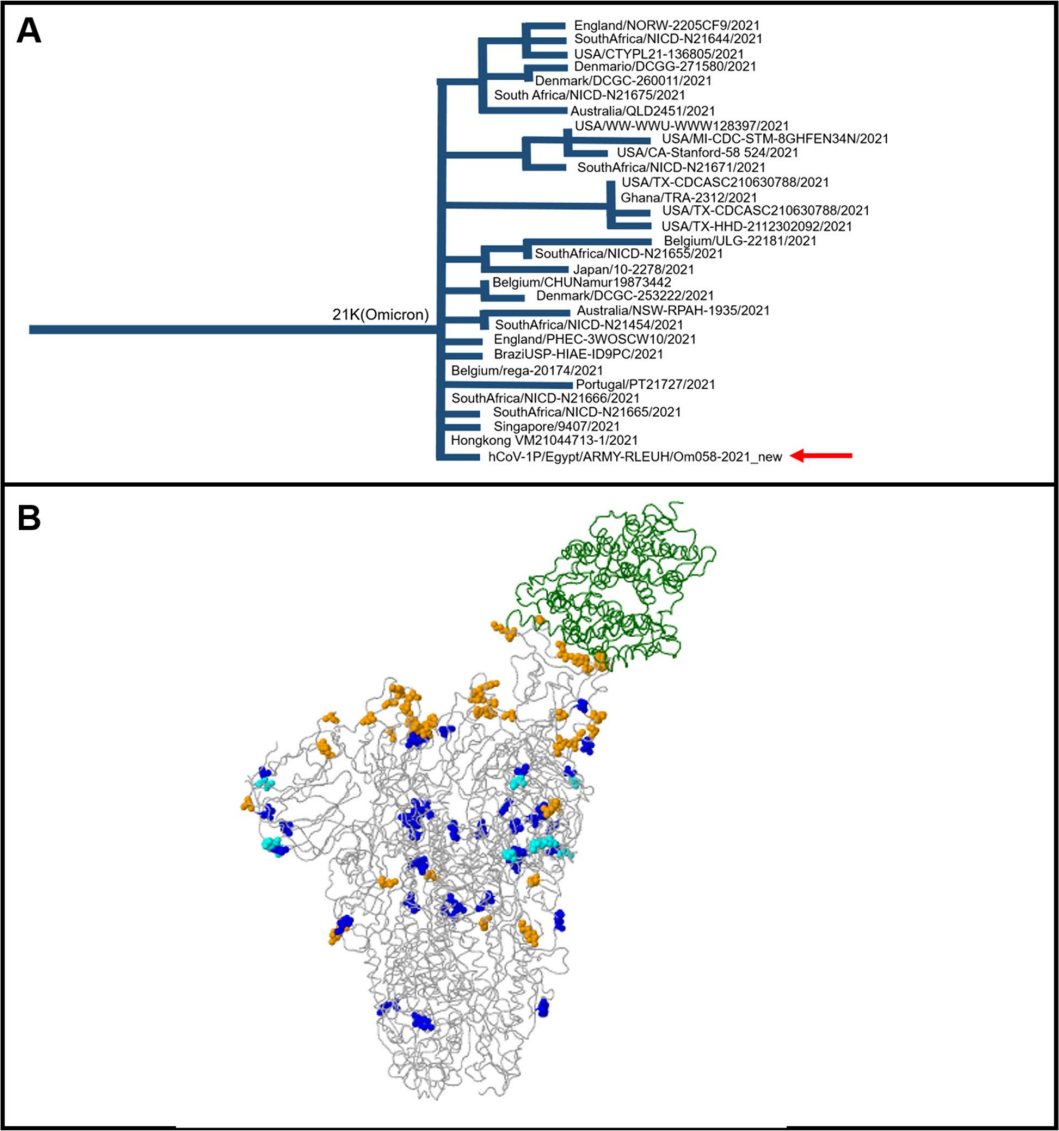
**A** Phylogenetic analysis of the omicron variant isolated in this case report. The isolate (arrow) is grouped inside the 21K sequences of SARS-CoV-2 virus. **B** structural visualization of the spike glycoprotein with aa changes identified in the query sequences shown as colored balls. Spike glycoprotein (PDB: 6acj, EM 4.2 Angstrom) in complex with host cell receptor ACE2 (green ribbon). The variations displayed in structure (nearest residue if in loop/termini region): A67V H69del V70del(69) T95I G142D V143del Y144del(143) Y145del(143) N211del L212I ins214EPE G339D V367F S371L S373P S375F S477N T478K E484A Q493R G496S Q498R N501Y Y505H T547K D614G H655Y N679K(674) P681H(674) D796Y N856K Q954H N969K L981F

Nextclade web tool v.012.0 [17] was used to compare the assembly sequence to SARS-CoV-2 reference sequences and to assign it to clades. As of 08 January 2022, the database includes 18 major clades. The variant is grouped inside the 21K clade (Fig. 1A). The public database GISAID (Shu and McCauley, 2017) was used for BLAST searches and for mutation analysis. The isolated sample showed the highest similarity to EPI_ISL_8427947 isolated in FL, USA; EPI_ISL_8427913 isolated in AL, USA; and EPI_ISL_8427762 isolated in PA, USA.

The mutations found in the sample were compared with the GISAID reference strain hCoV-19/Wuhan/WIV04/2019 using the CoVsurver mutation analysis tool (https://www.gisaid.org/epiflu-applications/%20covsurver-mutations-app/), which provides geographic and temporal distributions of SARS-CoV-2 mutations (Fig. 1B).

## Conclusion

In USA, for the first week of December 2021, more than 99% of circulating SARS-CoV-2 variants were Delta variants. Since that date, the number of infections with the Omicron variant is increasing significantly. Studies are currently in process to understand how easily it might be transmitted and the effectiveness of current diagnostic tests, vaccines, and therapeutics against this variant. First reported cases of Omicron variant infection showed mild symptoms, and symptoms would be expected to be milder in vaccinated persons and those with previous SARS-CoV-2 infection than in unvaccinated persons [18]. The severity of infection with the Omicron variant will become better understood as additional cases are identified and investigated.

In the present study, Omicron variant was confirmed by molecular diagnosis (RT-PCR). Screening for the suggested presence of Omicron VOC can be performed by identifying S gene target failure (SGTF) [19]. Rapid survey of omicron variant is based on the screening for S-gene target failures in a polymerase chain reaction (PCR)-based diagnostic assays. The Omicron genome (lineage BA.1) contains the S gene deletion at positions 69–70 that are widely used in PCR tests [20]. In the case of Omicron, both the N and ORF1ab genes are detected (with Ct values <=30) but the S gene is not. The variant identification is confirmed by genomic sequencing. The rapid emergence of the SARS-CoV-2 Omicron variant confirms the importance of using genome sequencing systems along with the PCR detections currently used. It indicates the importance of information-sharing among global public health partners.

## Data Availability

The sequence has been deposited in the Global Initiative on Sharing All Influenza Data (GISAID) database with accession ID: EPI_ISL_7952324.

## Abbreviations

GISAID: Global Initiative on Sharing All Influenza Data
RT-PCR: Reverse transcriptase-polymerase chain reaction
RLEUH: Reference laboratory for University Hospitals
SARS-CoV-2: Severe acute respiratory syndrome coronavirus 2
SGTF: S gene target failure
ViPR: Virus pathogen database and analysis resource
VOC: Variants of concern
*B. altitudinis*: *Bacillus altitudinis*
CMC: Carboxymethyl cellulose
CMCase: Carboxymethyl cellulase
FPase: Filter-paper enzyme
*gry*B: Gyrase B subunit
*rpo*B: RNA polymerase β subunit
SCB: Sugarcane Bagasse
*pyc*A: Pyruvate carboxylase
